# Defining functional states and roles of microglia in neuropsychiatric disorders

**DOI:** 10.3389/fncel.2026.1798151

**Published:** 2026-05-20

**Authors:** Kinga Szydlowska, Renan Tivanello, Bozena Kaminska

**Affiliations:** Laboratory of Molecular Neurobiology, Nencki Institute of Experimental Biology of the Polish Academy of Sciences, Warsaw, Poland

**Keywords:** disease-associated microglia, mass cytometry, microglia heterogeneity, microglia plasticity, mood disorders, single-cell RNA sequencing

## Abstract

Microglia are myeloid cells of the central nervous system (CNS) that acquire a context-specific phenotype and adjust their functions to microenvironmental cues. They participate in immune signaling, synaptic remodeling, and circuit functions, and have emerged as key culprits in neurodevelopmental and psychiatric disorders such as depression, anxiety, autism spectrum disorder (ASD), and schizophrenia. We characterize and discuss different functional state of microglia defined by sc-omics approaches that bring a high resolution to cell functionalities. Subsequently, we review the evidence of microglial states, microglia-driven mechanisms and their impacts on development and progression of neuropsychiatric disorders. In affective mood disorders, chronic stress, glucocorticoid dysregulation, and peripheral inflammation drive microglial nefarious activation. This leads to excessive synaptic pruning, impaired neurotrophic support, glutamate excitotoxicity, and circuit dysfunction in mood-related brain regions, with strong modulation by circadian mechanisms and sex-dependent factors. In ASD, microglia adopt a hybrid activation state characterized by altered inflammatory signaling, dysregulated phagocytosis, and aberrant synaptic pruning, driven by genetic and epigenetic mechanisms, including TREM2, ARID1A, complement components, and calcium-dependent glial signaling, which together disrupt network connectivity and social behavior. In schizophrenia, genetic risk factors related to *C4* and *DISC1*, along with inflammatory and metabolic stress, promote excessive microglia-mediated synapse elimination, cytoskeletal and motility deficits, and secondary neuronal metabolic dysfunction, which correlate with cognitive and negative symptoms. These findings strongly position microglia as a hub and key determinants of CNS homeostasis whose context-dependent dysregulation links immune, genetic, and environmental risk factors to synaptic and behavioral pathology. We discuss which microglial signaling pathways are shared and identify promising therapeutic targets across the neuropsychiatric disease spectrum.

## 1 Introduction

### 1.1 Microglia—versatile cells that are hubs for environmental clues

Microglia are the predominant myeloid cells of the central nervous system (CNS) which together with CNS border-associated macrophages (BAMs, now called CAMs—CNS-associated macrophages) play a crucial role in maintaining homeostasis and preventing CNS inflammation and injury (Prinz et al., 2021; Depp et al., 2025). Microglia reside in the CNS parenchyma, while CAMs are a distinct population and are located within CNS interfaces, such as the perivascular spaces (perivascular macrophages, PVMs), the leptomeninges (meningeal macrophages, MMs), and the choroid plexus (choroid plexus macrophages, ChPM). They are identified in human and mouse brain as CD163^+^ and CD206^+^ cells, respectively (Prinz et al., 2021). The origin and identity of microglia have been debated for many years due to numerous controversies and conflicting results (Prinz et al., 2021). Recent advances in single-cell technologies, such as single-cell or single-nuclei RNAseq (scRNA or snRNA-seq) along with Cite-seq (Cellular Indexing of Transcriptomes and Epitopes by Sequencing), single-cell mass spectrometry (cytometry by time-of-flight -CyTOF), and genetic fate mapping had provided new insights and tools unraveling microglia functionalities in diseases, which challenged many previous conclusions and concepts (Masuda et al., 2020). ScRNA-seq or snRNA-seq in human tissues enabled the identification of microglia by analyzing their transcriptional signatures while CyTOF with numerous surface markers permits the characterization of immune cell populations in humans and rodents.

The distinction of microglia from other tissue macrophages was first revealed by a single-cell transcriptomics study (using direct RNA sequencing), which demonstrated that *P2ry12*, *Tmem119, Gpr34, CSF1R, Siglech, Cx3cr1, Selpig, Ly86, Hexb*, *P2ry13*, *P2ry6*, and *Trem2* were highly expressed in microglia compared with peripheral macrophages but share genes typical for macrophages (*Cd11b*, *Cd14*, *Cd68*, *Tlr2* and *Tlr7*, *Cxcl16*, genes encoding various Fc receptors) (Buttgereit et al., 2016; Butovsky et al., 2014; Mrdjen et al., 2018). Interestingly, the majority of adult microglia expressed homeostatic genes with remarkably similar transcriptomes across brain regions. Early postnatal microglia are more heterogeneous, with a proliferative-region-associated microglia (PAM) subset occurring mainly in developing white matter, which shares a part of the gene signature with degenerative disease-associated microglia (DAM), has amoeboid morphology, is metabolically active, and phagocytoses new oligodendrocytes (Li et al., 2019). The CYTOF study confirmed the expression of P2Y_12_ and TMEM119, the high levels of CD64, CX3CR1, TGF-β1, TREM2, CD115, CCR5, CD32, CD172a, and CD91, and the low to absent expression of CD44, CCR2, CD45, CD206, CD163, and CD274/PD-L1 proteins in human microglia from autopsies (Böttcher et al., 2019). Gene ontology (GO) analysis classified the transcripts into pattern recognition receptors (25%), chemoattractant and chemokine receptors (10%), Fc receptors (7%), purinergic receptors (8%), receptors for extracellular matrix (ECM) proteins (6%), cytokine receptors (10%), receptors involved in cell–cell interaction (10%), other receptors or transporters (13%), and potential sensome proteins with no known ligands (11%). Notably, 32% of transcripts codes for the microglial sensome (*Entpd1*, *Tgfbr2*, *Cmtm7*, *Ly86*, *Cd180*, *Slco2b1*, *Gi24*, and *Clec4a2*) (Böttcher et al., 2019). A broad repertoire of cell surface receptors permits microglia dynamically survey and interact with their local environment (Hickman et al., 2013; Masuda et al., 2019). These transcriptional patterns are consistent with known functions of microglia in development, such as selective ECM and brain remodeling via engulfment of synapses and myelin (Stevens et al., 2007; Schafer et al., 2012) and highlight their potential to sense and recognize a plethora of microenvironmental clues. Human microglia (CD11b^+^CD45^Low^CD64^+^CX3CR1^High^ cells) display relatively well-conserved transcriptomic and epigenomic phenotypes as murine microglia. The intersection of the microglia gene signature with GWAS and transcriptomic data indicates that microglia are both responders and contributors to disease phenotypes (Gosselin et al., 2017).

Fate mapping with genetic engineered mouse models (Cx3cr1^CreERT2^, P2ry12^CreERT2^, Tmem119^CreERT2^, and Hexb^CreERT2^ mouse lines) allowed for more specific manipulation or visualization of microglia. Some of models (Cx3cr1^CreERT2^) could also trace CAMs expressing Cx3cr1. This obstacle is overcome with a binary transgenic model that co-expresses Sall1 [a solely microglia marker and Cx3cr1, specifically targeting microglia in a non-inducible manner (Kim et al., 2021)]. Fate mapping in mice showed that microglia and potentially CAMs arise from c-Kit+ noncommitted erythromyeloid progenitors (EMPs) that emerge around embryonic day 7.25 (E7.25) in the yolk sac (Ginhoux et al., 2010; Gomez Perdiguero et al., 2015). In humans, microglial precursors invade the brain primordium around 4.5–5.5 gestational weeks (Andjelkovic et al., 1998). During development, microglia begin to express classical microglial markers, such as *Sall1*, *Cx3cr1*, *P2ry12*, and *Selplg*, with peak expression in adulthood. Mass cytometry studies demonstrated that human microglia expressed higher mean levels of P2Y_12_, TMEM119, EMR1 (F4/80), CD64, and TREM2, whereas CD44, CCR2, CD45, CD14, and CD16 levels were much lower compared with the peripheral leukocytes and CSF (cerebrospinal fluid) cells (Böttcher et al., 2019).

Microglia are long-lived cells, and their proliferation rate is low in adulthood under physiological conditions. They renew slowly, with negligible input from circulating monocytes (Menassa et al., 2022). In adulthood, microglia depend on stimulation via colony stimulating factor 1 (Csf1) receptors. In both mice and humans, interleukin-34 (IL-34), an alternative ligand for Csf1 receptor produced by neurons in the brain, is essential for microglia maintenance. Multiple lineage tracing, parabiosis experiments, and scRNAseq analyses showed that all repopulated cells originate from the residual precursor microglia (Elmore et al., 2014; Luczak-Sobotkowska et al., 2024). Repopulated microglia recapitulate the heterogeneity of the naïve brain, and precursor cells transiently upregulate inflammatory gene expression and exhibit lower expression of homeostatic genes (Luczak-Sobotkowska et al., 2024).

### 1.2 Contextual clues drive functional and morphological microglia diversity

We used terms such as activation, state, phenotype, and plasticity in the specific context. While state and phenotype are interchangeable, activation means that microglia are expressing new markers/genes or downregulate typically for homeostatic microglia. In many instances activation is linked to morphological changes of microglia and its high motility. A term “plasticity” is used to highlight microglia propensity to adaptation and highlight that specific functional states depending on the context are typically a transient state. During homeostasis, microglia are highly branched cells that tightly tile all regions of the brain. A broad repertoire of cell surface receptors enables them to constantly survey their local environment (Hickman et al., 2013). Microglia respond to changes in their microenvironment across the lifespan and in disease conditions by adopting context-dependent states that result from epigenomic, transcriptomic, proteomic, and metabolomic features. All features combined result in discrete morphological, ultrastructural, and/or functional phenotypes (Paolicelli et al., 2022). scRNA-seq studies of myeloid cells across species-human, macaque, marmoset, sheep, mouse, hamster, chicken, zebrafish-revealed the evolutionarily conserved microglia core program, which includes genes involved in microglia maturation (*Sp1*, *Irf8*, *Tgfbr2*, *Csf1r*), linked to homeostatic functions (*C1qc*, *P2ry12*), and those encoding lysosomal hydrolases cathepsins (*Cst3*, *Ctsa*, *Ctss*, *Ctsb*, *Ctsh*, *Ctsc*, *Ctsz*, and *Hexa*), which are likely linked to phagocytotic functions. *Spp1*, *C3*, and *Vsig* were expressed mostly in large mammals, whereas expression of *Ccr5*, *Fcrls*, and *Siglech* was higher in mouse brains across various strains. Clusters specific to humans, macaque, marmoset, and sheep, when compared with rodents, revealed the enrichment of DNA repair, apoptotic cell clearance, and the complement pathway (Geirsdottir et al., 2019).

Key factors that lead to microglial functional heterogeneity include age, sex, circadian time, local CNS signals, changes in the microbiota, and the pathophysiological CNS states (Thion et al., 2018; Erny et al., 2015; Chen and Colonna, 2021; Silvin et al., 2022). Comparative studies of myeloid cell populations in the experimental autoimmune encephalomyelitis (EAE), the R6/2 model of Huntington’s disease (HD), and the superoxide dismutase 1 (mSOD1) model of amyotrophic lateral sclerosis (ALS) using single-cell mass cytometry identified three myeloid cell populations exclusive to the CNS and existing in each disease model. Blood-derived monocytes were prominent in EAE, but not in HD and ALS models.

Single-cell/nuclei transcriptomics of myeloid cells from different CNS pathologies and animal disease models showed that microglia tend to downregulate homeostatic genes while upregulating a transcriptional program characteristic of disease-associated microglia (DAM) in mice and humans (Deczkowska et al., 2018). DAM express core microglial markers: *Iba1*, *Cst3*, and *Hexb*, but expression of “homeostatic” microglial genes (*P2ry12*, *P2ry13*, *Cx3cr1*, CD33, and *Tmem119*) is lower, and genes such as *Apoe*, *Ctsd*, *Lpl*, *Tyrobp*, and *Trem2*, involved in lysosomal, phagocytic, and lipid metabolism pathways (Deczkowska et al., 2018), are upregulated (Keren-Shaul et al., 2017). Immunohistochemistry and smFISH (single-molecule FISH) studies in mice and human AD brain slices showed that DAM is spatially associated with AD lesions, and increases in lipid metabolism and phagocytosis-related genes may reflect a higher demand for plaque clearance. Massively Parallel Single-Cell RNA-seq (MARS-seq) analysis of DAM in Tg-AD and triggering receptor expressed on myeloid cells 2 (Trem2)^−/−^ Tg-AD revealed that the activation of the DAM program is initiated in a Trem2-independent manner, involving downregulation of microglial checkpoints, followed by activation of a Trem2-dependent program. A similar DAM subpopulation was found in an ALS mouse model (Keren-Shaul et al., 2017). The murine DAM and interferon response (Mathys et al., 2017) signatures were present in human microglia from AD patients. Antigen-presenting cells were enriched for AD genes that are downregulated in cortical tissues in both pathologically defined AD and AD dementia (Olah et al., 2020). Altogether, the evidence indicates a conserved “core DAM” signature in disease and aging characterized by the downregulation of the homeostatic microglia signature (*P2ry12*, *Cx3cr1*, and *Tmem119*) and induction of *Gpnmb*, *ApoE*, *Cst7*, *Fabp5*, *Lyz2*, *Ctsb*, *Clec7a*, *CD9, Lpl*, *Itgax*, *Igf1*, *Csf1*, and *Spp1*. However, changes in gene expression in human microglia depend on the specific pathology and its stage (Tuddenham et al., 2024). Additionally, environmental factors, including stress, diet, sleep patterns, physical activity, and microbiota composition, may impact microglia biology (reviewed in Madore et al., 2020).

### 1.3 The factors and cytokines instructive in shaping microglial functional states

Microglia can sense numerous signals via various surface and intracellular receptors (reviewed in Escoubas and Molofsky, 2024). They can sense neuronal firing via different chemical signals associated with it, such as the movement of sodium and potassium ions, the release of neurotransmitters like glutamate, and the release of purine compounds like ATP, ADP, and adenosine. Collectively, those receptors in microglia are known as receptors for brain-associated molecular patterns (BAMPs) and modulate neuronal activity. These include K^+^ (THIK-1), ATP (P2Y12), γ-aminobutyric acid (GABA), serotonin (5-HT_2B_), dopamine (DA), and noradrenaline (NE) (β_2_-AR) receptors (reviewed in Escoubas and Molofsky, 2024). Neuromodulators such as histamine, norepinephrine, dopamine, serotonin, and acetylcholine are important modifiers of neural network activity, secreted into the extracellular space and acting over micrometers. Other factors include myelin, extracellular matrix (ECM) (Nguyen et al., 2020), and dsRNA (Town et al., 2006). Microglia are phagocytic cells of the CNS and eliminate damaged or dead cells by recognizing phosphatidylserine (PS), a major component of the lipid bilayer, which is exposed on the outer layer in dying cells. The modified surface molecules ICAM-3 and CD31 act as “eat me” signals (Grimsley and Ravichandran, 2003). The G protein-coupled receptor GPR56 and MER proto-oncogene tyrosine kinase (MerTK) receptor are proposed to be potential microglial receptors for exposed PS, as genetic deletion of these receptors in microglia prevented synapse phagocytosis (Park et al., 2021). CD47 (a transmembrane immunoglobulin Ig superfamily protein) acts as a “don’t eat me” signal, and with its receptor, SIRPα, they are negative regulator of microglia-mediated synapse pruning during brain development (Lehrman et al., 2018). Interplay between “eat me” and “do not eat me” signals, such as complement and CD47, instructs phagocytes in the immune system on what to engulf without triggering local inflammation (Grimsley and Ravichandran, 2003).

Cytokines and chemokines are means of cell-to-cell communication and cytokine receptors are broadly expressed across myeloid cells. Cytokines instructing microglial functional states include interleukins and interferons. IL-1 family cytokines are structurally related cytokines that enhance innate immune responses, and share some signaling proteins with Toll-like Receptors (TLRs). Interleukin-33 (known as an “alarmin”) signals to microglia in excitatory synapse remodeling likely by ECM remodeling. Deletion of IL-33 or its receptor IL1RL1 in microglia, showed the role of this signaling in microglial phagocytosis and excitatory synapse density regulation. Microglia express IL1R1, the receptor for IL-1α and IL-1β, along with the decoy receptor IL1-R2 (an endogenous negative regulator). *Il1r1* deficient mice show reduced microglial phagocytosis, increased synapse density in the developing hippocampus, and some behavioral alterations. Type I interferons (IFN-α/β) are locally produced and signal through the IFNAR1 receptor, while type II interferons (IFN-γ), which are produced by lymphocytes, signal through IFNGR1/2. Loss of IFN-I signaling by knockout of its receptor (*Ifnar1^−/−^*) led to an atypical “bubble” morphology with swollen phagosomes in 40% of microglia, associated with the appearance of excitatory neurons with DNA-damage and tactile hypersensitivity in the offspring (reviewed in Escoubas and Molofsky, 2024). Microglia express numerous other cytokine and chemokine receptors, such as CX3CR1, but most of them have not been studied in homeostatic settings *in vivo*.

## 2 Microglia states, roles and underlying signaling in neuropsychiatric disorders

### 2.1 Microglia activation in neuropsychiatric disorders

There is growing evidence that activated microglia contribute to pathophysiology in affective disorders such as major depressive disorder, schizophrenia, anxiety, and autism spectrum disorder, affecting brain homeostasis, neural connectivity, and neurotransmitter systems (Gandal et al., 2018; Wang et al., 2022). In this review, we will discuss the molecular mechanisms by which stress or early maternal immune activation primes microglia toward an active, slightly pro-inflammatory state. We aim to identify unique and shared mechanisms underlying microglial activation in various mood disorders. By integrating insights across various models, the review will provide a comprehensive perspective on the roles of phagocytic and inflammatory microglial cells in different disorders, underscoring both mechanistic understanding and therapeutic potential for neuropsychiatric disorders.

### 2.2 Microglia in affective disorders

Affective disorders such as major depressive disorder (MDD) and anxiety-related conditions represent an important global health challenge, with anxiety disorders alone affecting more than 300 million people worldwide (Baxter et al., 2014). These disorders are characterized by depressed mood, diminished interests (anhedonia), impaired cognitive function, and excessive worry, fear, or apprehension (Filiou and Sandi, 2019; Rahman and Alzarea, 2019; Li et al., 2013). Disturbances in sleep and appetite are also common among those diagnosed with MDD (Filiou and Sandi, 2019; Rahman and Alzarea, 2019; Li et al., 2013). The economic burden associated with depression is extremely high and, in the United States alone, is estimated at $210.5 billion (Greenberg et al., 2015). Epidemiological research consistently demonstrates strong links between the development of depression and early life and adulthood stress in individuals from different cultures, sexes, and ancestries (GBD 2016 Neurology Collaborators, 2019; Kuehner, 2017; Seedat et al., 2009). Women are disproportionately affected, with estimates of depression incidence indicating a 2-fold higher rate of depression in women than in men (GBD 2016 Neurology Collaborators, 2019; Kuehner, 2017; Seedat et al., 2009). Chronic stress is a primary trigger or exacerbator of MDD, and individuals with a higher susceptibility to stress exhibit a higher risk for MDD (Ding and Dai, 2019; Gulyaeva, 2021).

One of the hypotheses about the mechanisms of MDD is related to inflammation, as many depression patients suffer from increased activation of the immune system (Krishnan and Nestler, 2008). This immune activation is characterized by elevated levels of proinflammatory cytokines, specifically tumor necrosis factor (TNF)-α, interleukin (IL)-6, and IL-1β, both systemically and within the CNS (Fitzgerald et al., 2006). Trafficking of peripheral immune cells into the brain and the subsequent activation of resident microglia are part of the process (Brás et al., 2022). Applications of traditional antidepressants having anti-inflammatory properties have yielded contradictory results, and undergoing inflammation was aberrant in non-responders (Strawbridge et al., 2015; Haroon et al., 2018). A significant association between the number of failed antidepressant treatment trials and elevated levels of TNF-α, IL-6, and C-reactive protein was reported (Haroon et al., 2018). Treatment with pro-inflammatory cytokines (e.g., TNF-α, IL-1, IL-6, interferon IFNα) triggered neuroinflammation, disrupted neurotransmitter metabolism, and activated the hypothalamic–pituitary–adrenal (HPA) axis, which induced MDD symptoms such as fatigue, anxiety, and anhedonia (Felger and Lotrich, 2013; Raison et al., 2013; Weinberger et al., 2015). Diverse antidepressants consistently demonstrate immunomodulatory properties that extend beyond their effects on neurotransmitters and reduce pro-inflammatory markers (IFN-γ, TNF-α, IL-6) while augmenting anti-inflammatory cytokines (IL-10, transforming growth factor (TGF)-β) (Nava et al., 2025). Clinical trials using cytokine antagonists have demonstrated efficacy in improving mood and behavior, particularly in treatment-resistant patients with elevated inflammatory biomarkers (Raison et al., 2013; Weinberger et al., 2015).

Chronic stress induces remodeling of microglial morphology (Paolicelli and Ferretti, 2017; Bassett et al., 2021; Torres-Platas et al., 2014), which leads to dysregulated interactions with neurons (Paolicelli and Ferretti, 2017; Bassett et al., 2021; Torres-Platas et al., 2014). Neuroimaging studies in depressed patients have shown that symptom severity correlates with microglial activation in brain regions involved in mood regulation, such as the prefrontal, anterior cingulate, and insular cortices (Setiawan et al., 2015). Additionally, individuals exposed to chronic stress show an increased production and release of proinflammatory monocytes into circulation. Circulating Ly6Chi monocytes are recruited into specific brain regions, promoting neuroinflammation and anxiety (Heidt et al., 2014; Cole et al., 2011). In the medial prefrontal cortex (PFC), chronic unpredictable stress (CUS) causes synaptic loss in pyramidal neurons and increased branching of microglia (Salter and Beggs, 2014; Kettenmann et al., 2013; Hinwood et al., 2013).

The role of glucocorticoids (GCs), especially cortisol, is central to the development of mood disorders (Fukumoto et al., 2009; Oliveira et al., 2012; Tanokashira et al., 2012; Gilman et al., 2013; McLaughlin et al., 2010). This is particularly critical when exposure occurs above physiological levels during early life or adulthood (Fukumoto et al., 2009; Oliveira et al., 2012; Tanokashira et al., 2012; Gilman et al., 2013; McLaughlin et al., 2010). Early-life stress exposure and elevated cortisol levels significantly enhance an animal’s susceptibility to depressive-like behaviors in adulthood (Brás et al., 2022). Specifically, stressful early-life events combined with high cortisol stress responsiveness lead to neuro-immunological alterations, including increased TNF-α levels and microglia activation (Brás et al., 2022). Exposure to high GC levels during development causes deleterious structural changes in the brain, such as impaired neuronal migration, altered spine formation, and reduced neuronal survival (Caetano et al., 2017). Prenatal exposure of mice to the synthetic GC dexamethasone (DEX) triggers a long-lasting, sex-specific remodeling of microglial processes in the PFC, which correlates with anxiety phenotypes (Fukumoto et al., 2009; Oliveira et al., 2012; Tanokashira et al., 2012; Crochemore et al., 2005). Clinical reports show that subsets of depressed individuals have elevated blood levels of cortisol (Gold, 2015; Lamers et al., 2013; Stetler and Miller, 2011). Preclinical studies indicate that both GC signaling and chronic stress can induce dendritic atrophy and synaptic deficits in pyramidal neurons in the medial PFC (Liu and Aghajanian, 2008; Radley et al., 2008; Wellman, 2001). This process is mediated by neuron–microglia interactions, in which chronic stress-induced GC signaling increases neuronal colony stimulating factor (CSF) 1 expression, which, in turn, triggers neuronal remodeling by microglia (Wohleb et al., 2018). Antagonizing GC receptors with agents such as RU486 can prevent the onset of behavioral issues, limit microglial remodeling, and mitigate synaptic deficits following chronic stress, indicating a causal role for these hormones in pathology (Horchar and Wohleb, 2019).

A major regulator of neuroinflammation is the P2X7 receptor (P2X7R), an ATP-gated ion channel primarily found on activated microglia (Hong et al., 2020). Activation of P2X7R facilitates the release of ATP and inflammatory cytokines into the extracellular space and directly regulates hippocampal glutamate release (Ribeiro et al., 2019). This receptor also influences the signaling pathway of brain-derived neurotrophic factor (BDNF), which is crucial for the therapeutic actions of antidepressants (Ribeiro et al., 2019). Genetic studies have linked highly polymorphic *P2X7R* genes and single-nucleotide polymorphisms (SNPs) to an increased risk of developing depression and anxiety in humans (McQuillin et al., 2009; Roger et al., 2010). In animal models of repeated social defeat (RSD), blocking P2X7 activation with an antagonist JNJ-54471300 attenuates neuroinflammation in the hippocampus and amygdala, and reduces anxiety-like behavior (Biltz et al., 2024). Preclinical studies using lithium-pilocarpine-induced epileptic rats found that P2X7R expression is notably increased in activated microglia (Hong et al., 2020). Treatment with the P2X7R antagonist (brilliant blue G) attenuated depression- and anxiety-like behaviors as effectively as the antidepressant fluoxetine while inhibiting neuron loss in the hippocampus (Hong et al., 2020).

Circadian rhythms in mammals are controlled by “clock” genes, including *Bmal1, Clock,* and *Rev-erbα* (Partch et al., 2014). Disruption of the circadian system is strongly associated with mood disorders. In humans, circadian patterns of gene expression are much weaker in the brains of individuals suffering from MDD (Li et al., 2013). Rev-erbα knockout (RKO) mice display high risk-taking, aggression, cognitive deficits, and a mixed anxiety-depressive phenotype (Chung et al., 2014; Jager et al., 2014; Otsuka et al., 2022). These behavioral changes are accompanied by increased neuroinflammation in the hippocampus, characterized by increased microglia counts and pro-inflammatory cytokine expression (Chen et al., 2023). Rev-erbα deficiency may increase activity in phagocytosis-related pathways, as evidenced by colocalized expression of CD68 and Iba1, contributing to defects in neuronal activity (Chung et al., 2014; Jager et al., 2014; Otsuka et al., 2022). Microglia can regulate neuronal activity by affecting synaptic pruning and phagocytosis in a circadian manner, and their depletion with the CSF1R inhibitor PLX5622 ameliorates neuroinflammation and some behavioral deficits in RKO models (Chen et al., 2023; Partch et al., 2014).

The fractalkine (CX3CL1-CX3CR1) signaling is a unique communication channel through which neuronal cells expressing CX3CL1 regulate microglia activation (Cardona et al., 2006; Hughes et al., 2002; Maciejewski-Lenoir et al., 1999; Pan et al., 1997). Deletion of the CX3CR1 receptor increases the inflammatory profile in the brain and is associated with decreased neurogenesis and deficits in motor and spatial learning (Cardona et al., 2006; Hughes et al., 2002; Maciejewski-Lenoir et al., 1999; Pan et al., 1997). After an acute inflammatory challenge with lipopolysaccharide (LPS) CX3CR1 KO mice exhibit prolonged neuroinflammation and depressive-like behavior, associated with indoleamine 2,3-dioxygenase (IDO)-mediated tryptophan catabolism into neurotoxic metabolites (Corona et al., 2013). Inhibition of IDO with 1-Methyl-tryptophan (1-MT) attenuates microglia activation in the PFC (prefrontal cortex) and hippocampus, and blocks the appearance of depression symptoms (Corona et al., 2013). In the chronic unpredictable mild stress depression model, the protein Dkk3 is decreased in both neurons and microglia in the hippocampal CA1 region (Partch et al., 2014). Neuronal-specific knockdown of Dkk3 (Dickkopf-3, a secreted glycoprotein) promotes microglial phagocytosis and depressive symptoms by regulating the Wnt-CX3CL1/CX3CR1 signaling pathway (Partch et al., 2014). Treatment with monoclonal antibodies against CX3CL1 or targeting the Wnt (Wingless/Integrated) signaling pathway has shown potential to reduce stress-induced symptoms (Partch et al., 2014).

The basolateral amygdala (BLA) is a crucial brain region for integrating sensory information and assigning affective value to it. It influences anxiety and depression (Janak and Tye, 2015; Heshmati and Russo, 2015; Lalumiere, 2014). Acute LPS challenge induced increased Iba1 immunoreactivity and pro-inflammatory cytokine production in the BLA (Zheng et al., 2021; Li et al., 2017; Mello et al., 2013). This neuroinflammatory response led to hyperactivity of BLA projection neurons, characterized by enhanced glutamatergic synaptic transmission and increased excitability (Zheng et al., 2021). Such dysfunctions contribute to the pathophysiology of psychiatric disorders and can be reversed by fluoxetine pretreatment (Zheng et al., 2021). Fluoxetine treatment has been shown to prevent the development of anxiety- and depressive-like behaviors by the inhibition of microglia activation in both cultured cells and animal models of peripheral inflammation (Zheng et al., 2021).

Recent single-cell analyses have challenged the traditional neuroinflammation hypothesis of MDD, suggesting a non-inflammatory microglial phenotype characterized by enhanced homeostatic functions. CyTOF studies of post-mortem brain tissue showed that microglia in individuals with MDD exhibit significantly increased expression of homeostatic markers, specifically the P2Y12 receptor (P2RY12), transmembrane protein 119 (TMEM119), and the chemokine receptor CCR5 (CD195) (Böttcher et al., 2020). These cells also showed a marked downregulation of classic activation markers, such as HLA-DR and the lysosomal protein CD68. This shift toward a “hyper-homeostatic” state is further supported by the absence of proinflammatory cytokine induction, with no significant changes observed in genes encoding IL-1β, IL-6, TNF, CCL2, and MIP-1β (CCL4) within microglial clusters (Böttcher et al., 2020). Additionally, MDD brains show an enrichment of non-inflammatory CD206hi macrophages, suggesting a broader trend toward immune suppression or altered surveillance rather than active inflammation (Böttcher et al., 2020).

Studies using single-nucleus chromatin accessibility profiling identified a specific gray matter microglia cluster, termed Mic2, which exhibits significantly reduced chromatin accessibility (reflecting reduced gene expression) in individuals with MDD (Chawla et al., 2025). This cluster shares molecular characteristics with previously identified “depression disease-associated microglia” (depDAM), which are defined by the downregulation of genes involved in immune response and phagocytosis (Scheepstra et al., 2023). The reduced accessibility in Mic2 is particularly prominent at binding sites for transcription factors critical for maintaining immune homeostasis and lineage specificity, such as PU.1 (SPI1) and various interferon regulatory factors (IRFs), including IRF1. Regulatory networks linked to these differentially accessible regions involve genes such as *CD74, CD86, CEBPA,* and *ALOX5AP* (Chawla et al., 2025). Furthermore, genetic risk variants for MDD, such as the SNP rs10210757, have been shown to disrupt the binding sites of transcription factors like FLI1, potentially impacting the regulation of genes such as *ARL4C*, which is involved in lipid homeostasis within immune cells (Chawla et al., 2025). These findings suggest that MDD pathology involves a complex dysregulation of microglial gene-regulatory mechanisms that favor a suppressed or altered homeostatic state over a classic inflammatory response.

Appreciation of a central role of microglia in the risk and progression of depression and anxiety directs toward specific therapeutic strategies (Yirmiya et al., 2015; Rimmerman et al., 2022; Yao et al., 2022; Mariani et al., 2022). Various treatments, including electroconvulsive therapy, ketamine, and standard selective serotonin reuptake inhibitors (SSRIs), modulate microglia functions as part of their antidepressant response (Yirmiya et al., 2015; Rimmerman et al., 2022; Yao et al., 2022; Mariani et al., 2022). Drugs such as minocycline, a well-described modulator of microglial function, exhibit antidepressant effects in chronic stress models and prevent monocyte recruitment (Bassett et al., 2021; McKim et al., 2018). Other pathways, such as the STING (Stimulator of Interferon Genes) adaptor protein (Ishikawa and Barber, 2008; Konno et al., 2013) which links DNA sensors, offer novel targets. The STING agonist 2′3-cGAMP induces a type 1 interferon response (IFN-β) that enhances microglial phagocytosis and decreases neuroinflammation (Duan et al., 2022; Konno et al., 2013; Ishikawa and Barber, 2008). Additionally, STAT3 signaling in microglia is essential for promoting antidepressant-like behavior by regulating M-CSF effects on synaptic transmission via ERK1/2 and Akt/GSK3B pathways (Kwon et al., 2017; Dwivedi et al., 2001; Dwivedi et al., 2006; Duman et al., 2012; El Kasmi et al., 2006; Karege et al., 2007).

**Figure 1:**
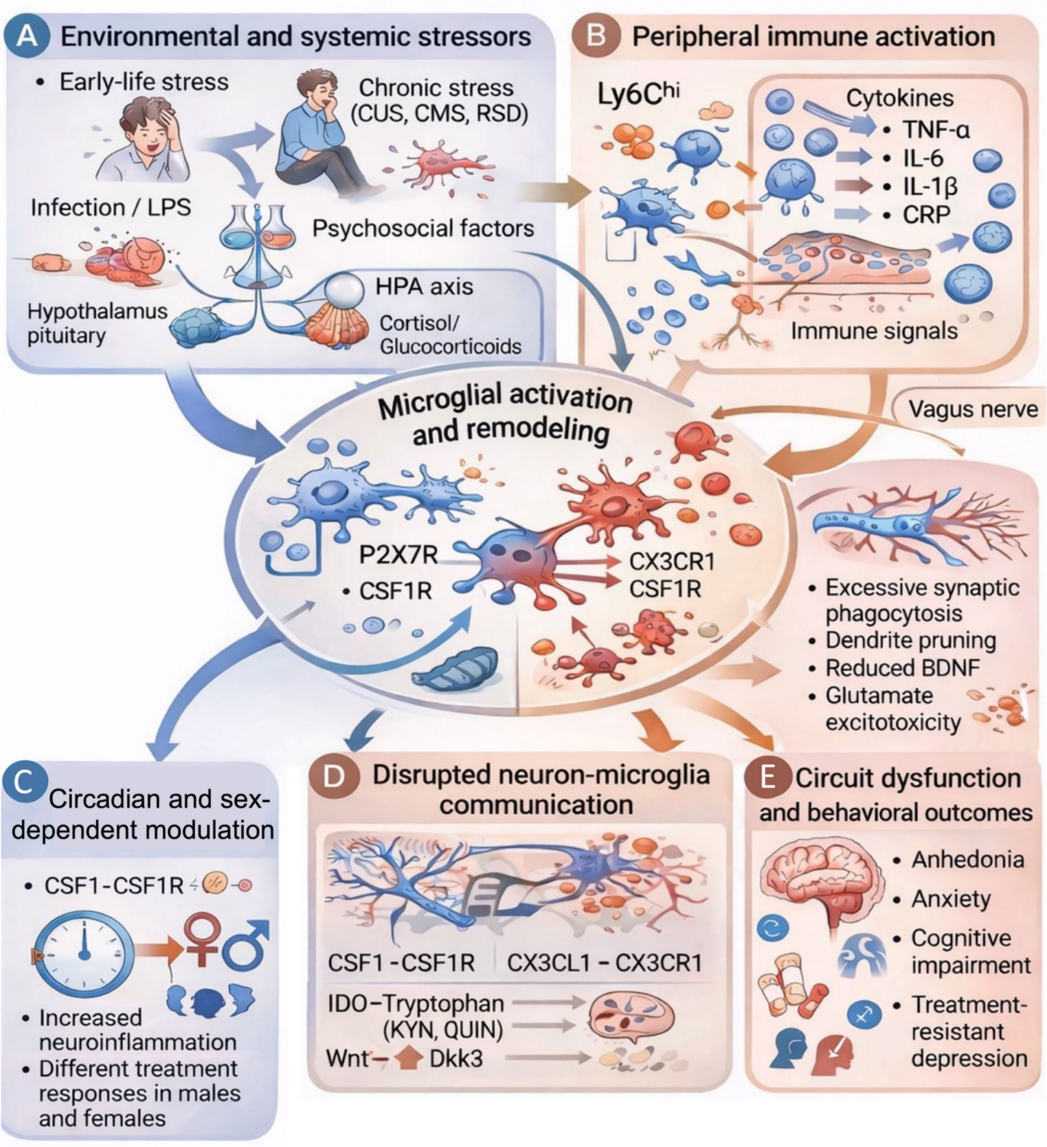
Microglia as critical mediators of neuroplasticity in affective disorders.

In summary, microglia are activated in mood disorders and mediate aberrant neuroplasticity. Microglia dysregulation is driven by cytokines, lipids, purinergic signaling, and clock genes, and contribute to the pathophysiology of mood disorders. Interventions aimed at normalizing neuron–microglia interactions via microglia-related pathways such as CSF1, P2X7R, or CX3CR1 offer promising strategies for treating stress-related affective disorders (Wohleb et al., 2018; Horchar and Wohleb, 2019; Partch et al., 2014) (Figure 1).

Graphics present neuroimmune mechanisms by which stress and inflammatory responses affect microglia in affective disorders. (A) Early-life stress, chronic psychosocial stress, and peripheral inflammation activate the hypothalamic–pituitary–adrenal (HPA) axis. This results in long-term elevations in glucocorticoid levels. (B) Stress exposure and immune activation promote peripheral inflammatory responses. They are characterized by increased circulating Ly6C^hi monocytes and elevated levels of pro-inflammatory cytokines, such as TNF-α, IL-6, and IL-1β. Pro-inflammatory signals induce microglial activation, leading to CNS remodeling via P2X7R, CX3CR1, and CSF1R signaling, mitochondrial dysfunction, and increased inflammatory responsiveness. (C) Microglial activity can be modulated by circadian clock genes (*Bmal1, Clock, Rev-erbα*) and exhibits sexual dimorphism. Differential neuroinflammatory profiles and different treatment responses are observed in males vs. females. (D) Activated microglia promote excessive synaptic pruning and phagocytosis. As a result, BDNF levels decrease and glutamate excitotoxicity increases. These processes are mediated by CSF1-CSF1R, CX3CL1-CX3CR1, IDO-dependent tryptophan metabolism, and Wnt/Dkk3 signaling. (E) Synaptic and circuit-level dysfunctions in the prefrontal cortex, hippocampus, and amygdala lead to behavioral changes like anhedonia, anxiety, cognitive impairment, and depression.

### 2.3 Microglia alterations in autism spectrum disorder

Autism spectrum disorder (ASD) is a neurodevelopmental condition characterized by difficulties in social communication and repetitive behaviors. Although ASD is a multifactorial disease involving both genetic and environmental factors, recent studies have reported that dysregulation of the immune system plays a role in its pathogenesis (Wamsley et al., 2024; Velmeshev et al., 2019; Edmonson et al., 2016). Microglia activation and neuroinflammatory responses are reported in ASD (Wamsley et al., 2024; Gupta et al., 2014). Postmortem studies of individuals with ASD showed increased microglia density and hypertrophy in some brain regions, including the cortex and cerebellum (Tetreault et al., 2012). Elevated levels of pro-inflammatory cytokines, such as interleukins and TNF-α, in brain tissues and cerebrospinal fluids were also reported (Petrelli et al., 2016; Ohja et al., 2018; Runge et al., 2023). Combining snRNA-seq and single-nucleus assay for transposase-accessible chromatin with sequencing (snATAC-seq) with spatial transcriptomics revealed genes typical for activated microglia and astrocytes in superficial gray matter layers, and somatostatin (SST) interneurons in superficial cortical laminae, along with intense down-regulation of synaptic gene expression and up-regulation of stress-response and proinflammatory pathways in inter- and intrahemispheric projection neurons. The integrated snRNA-seq, snATAC-seq, and spatial transcriptomics analysis revealed dysregulated gene regulatory networks (GRNs) in microglia, with target genes augmenting stress-response and proinflammatory pathways, while others regulate genes involved in phagocytosis or proliferation. These GRN and their genes are present in the superficial cortical laminae, indicating microglia activation (Wamsley et al., 2024). These processes may alter neuronal synapses (Ashwood et al., 2011; Gupta et al., 2014; Runge et al., 2023). Altogether, these data showed a complex interaction between metabolic stress and immune activation of microglia, which results in ASD symptoms.

These findings confirmed previous studies by Velmeshev et al., who used snRNAseq of postmortem brain tissue from different regions, showing that microglia from ASD donors adopt a transcriptomic profile distinct from controls, enriched for genes involved in activation, development, and immune signaling (Velmeshev et al., 2019). Studies by Gupta et al. also indicate a unique microglia state in ASD. They showed that neuronal and microglial genes were robustly dysregulated in the autism cortical brain in comparison with controls, and identified co-expression of microglial genes that resemble an anti-inflammatory activation signature and brain-derived neurotrophic factor (BDNF), which had a negative correlation with altered neuronal activity-dependent genes (Gupta et al., 2014). These data showed a dysregulated balance between innate immune activation and neuronal signaling (Gupta et al., 2014). Among the genes are immune response genes and chemokines, suggesting that microglia in ASD are associated with remodeling rather than classical inflammation-mediated cytotoxicity. Ohja et al. reported that microglia express interferon-stimulated genes (ISGs), reactive oxygen species genes, and factors in response to life stress (Ohja et al., 2018).

Reemst et al. (2022) found altered proportions of morphological subtypes of microglia in the hippocampus of adult mice exposed to early-life stress (ELS), along with transcriptomic changes related to the TNF response and protein ubiquitination (Reemst et al., 2022). ELS microglia showed reduced synaptosome phagocytic capacity. ELS had a different impact on gene expression profiles during microglial development from P9 to P200 and gene expression profiles differ from the response to an LPS challenge at P200. The ELS-induced increased expression of *GAS6* (the phagocytosis-related gene) in mice, and its increase was found in the dentate gyrus of individuals with a history of child abuse by *in situ* hybridization (Reemst et al., 2022). Therefore, these data converge on microglia in ASD occupying a unique state that includes stress response, immune activation, and phagocytic remodeling.

In ASD, microglia display upregulated pathway genes, such as *C1q* and *C3*, suggesting synaptic pruning. The *C1q* gene encodes the complement protein that initiates the classical cascade by binding to synapses that should be eliminated. This binding triggers the deposition of the C3 protein, more specifically the activated fragment C3b (Stevens et al., 2007). Microglia express the C3 receptor (CR3/Mac-1) which allows them to physically recognize and phagocytose the marked synapses. High expression of these genes led to excessive synaptic elimination and altered connections in ASD brains (Stevens et al., 2007).

Epigenetic regulators, such as chromatin remodeler ARID1A (AT-Rich Interaction Domain 1A), are involved in microglia homeostasis. Disruption of ARID1A-active microglia stimulates cytokine production, leading to social impairment in ASD (Su et al., 2024). The knockout of the *Aridla* gene in animal models resulted in a reduction of microglial homeostasis markers, such as P2RY12 and CDF4/80. The knockout also caused a global increase in the histone repressor mark H3K9me3, which directly silences the *proteoglycan 3* gene. The lack of the latter in microglia dysregulates the Wnt/β−catenin pathway in neural progenitors (NPCs) resulting in neurogenesis defect. Moreover, a lack of ARID1A resulted in activated microglia, as visualized by confocal microscopy, and the appearance of autistic behavior in the animal model (Su et al., 2024).

Recent scRNAseq studies have identified subtypes of ASD microglia, including DAMs characterized by dysregulation of TREM2 pathways (Tian et al., 2024; Filipello et al., 2018). TREM2^−/−^ mice exhibited connectivity and behavioral deficits associated with ASD (Filipello et al., 2018). TREM2-mediated pruning induces the pro-inflammatory protein CD86, while simultaneously reducing the anti-inflammatory protein CD206 (Tian et al., 2024). In mouse models with non-functional microglia, alterations in synaptic density and deficits in social interactions were observed (Paolicelli et al., 2011; Zhan et al., 2014). Furthermore, TREM2 deletion causes both an increase in p-38 MAPK levels and a reduction in p-ELK-1 levels, resulting in the activation of the anti-inflammatory phenotype. Normalization of TREM2 levels also reversed the microglial phenotype to anti-inflammatory. Moreover, some cytokines secreted by microglia, such as BDNF, TNF-α, and IL-33, also modulated synaptic transmission and induced social isolation behavior (Paolicelli et al., 2011; Han et al., 2023; Komori et al., 2024). Thus, TREM2 in microglia may modulate synaptic plasticity by eliminating inactive synapses.

Due to a bidirectional “glia–glia” signaling network, astrocytes and microglia can influence each other, and disruption in this communication can provoke neuroinflammation and affect synaptic homeostasis (Faust et al., 2025; Petrelli et al., 2016). The coordination between glia cells is regulated by microglia-derived Wnt ligands, which instruct astrocytes to retract their perisynaptic processes to permit subsequent synapse removal (Allen et al., 2022). Furthermore, the gene required for microglial Wnt secretion (*WLS*) is downregulated in the microglia of ASD patients, which evidence that a microglial transcriptional failure disrupts the communication for circuit refinement (Allen et al., 2022).

**Figure 2:**
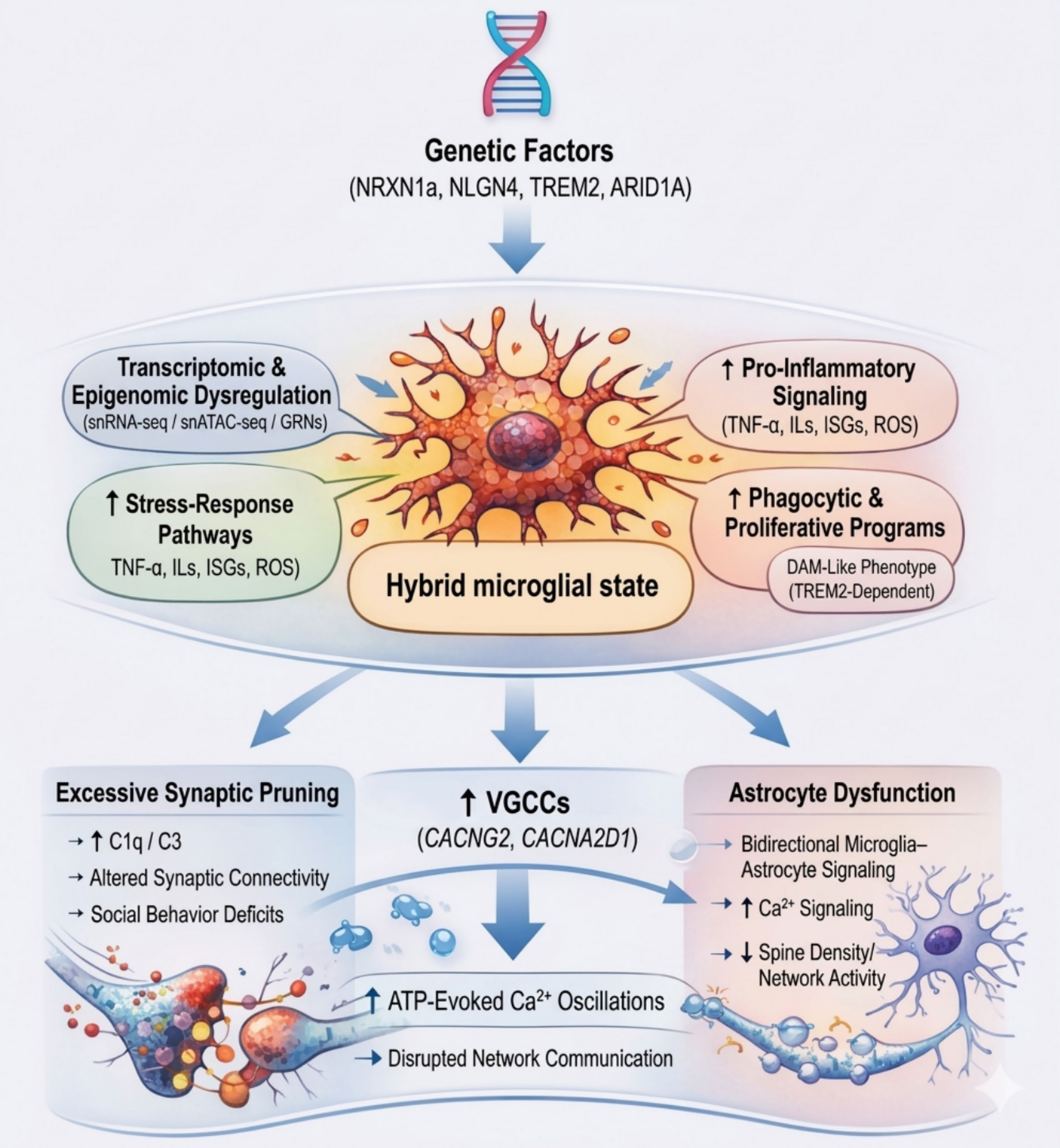
Microglia dysregulation and neuroimmune mechanisms in autism spectrum disorder.

In a translational study, Allen et al. generated human induced pluripotent stem cell (iPSC)-derived astrocytes from individuals with ASD, transplanted them into neonatal mouse brains, and observed effects on both behavior and synaptic physiology (Allen et al., 2022). These cells showed reduced spine density and neuronal network activity *in vitro* (Allen et al., 2022). Applications of calcium imaging and targeted genetic perturbation of the Ca^2+^ machinery demonstrated a link between elevated calcium signaling in glial cells and ASD phenotypes, specifically an increased amplitude and frequency of ATP-evoked Ca^2+^ transients. These results were confirmed in proteomic analyses, as elevated levels of Ca^2+^ proteins, and live-cell imaging showed increased amplitude and frequency of ATP-evoked Ca^2+^ signals (Allen et al., 2022). Altogether, those studies demonstrate that microglial dysfunction plays a central role in ASD pathology through mechanisms involving synaptic pruning, epigenetic dysregulation, and induced inflammatory processes. The upregulation of genes, such as *ARID1A* and *TREM2*, and the involvement of microglial subtypes contribute to unbalanced synapses and behavioral features of ASD (Figure 2).

Dysregulation of genes such as *ARID1A* and *TREM2* in autism spectrum disorder may alter microglia phenotypes and their interactions with other glial and neuronal cells. The main changes in ASD include increased microglial density and elevated levels of pro-inflammatory cytokines, which alter metabolism, dysregulated synaptic plasticity and excessive synaptic pruning. Dysfunctional communication between microglia, astrocytes, and neurons, modulated by Ca^2+^ signaling and inflammatory mediators, alters synaptic homeostasis and influences behaviors associated with ASD.

### 2.4 Role of microglia in schizophrenia

Schizophrenia (SCZ) is a chronic and severe neuropsychiatric disorder characterized by alterations in brain connections and functions. Experimental data indicate that mechanisms underlying SCZ pathology involve alterations in synaptic function and increased microglial-mediated inflammatory responses in the brain. Aberrant microglia activation is one of the hypotheses for SCZ pathogenesis and acquired a strong experimental support from recent studies (Gandal et al., 2018; Chen et al., 2022; Ruzicka et al., 2024; Franklin et al., 2025; Kessels et al., 2025). Recent studies suggest that in SCZ, genetic risk factors and molecular changes that impair microglial function result in downstream cellular and functional deficits that manifest as clinical symptoms (Kessels et al., 2025; Franklin et al., 2025; Shiwaku et al., 2022). The potential mechanisms have been unraveled by post-mortem tissue analysis, genetic association studies, and human induced pluripotent stem cell (hiPSC) derived neuron and microglia co-culture systems (Yao et al., 2022; Franklin et al., 2025; Torshizi et al., 2019; Ruzicka et al., 2024; Brennand et al., 2015).

Epidemiological studies have shown an association between maternal infection, microglia activation, and schizophrenia or autism in the progeny. Studies in animal models demonstrated that maternal immune activation (mIA) is a profound risk factor for neurochemical and behavioral alterations in the offspring (Knuesel et al., 2014). A hypothesis linking immune dysfunction to structural changes in SCZ involves the classical complement cascade, including the complement component 4 (C4) locus (Sellgren et al., 2019; Yilmaz et al., 2021; Breitmeyer et al., 2023). The *C4* gene is a risk factor for SCZ in humans, promoting excessive synaptic loss and behavioral changes (Yilmaz et al., 2021; Breitmeyer et al., 2023). The C4A isoform is more efficient than C4B at marking synapses for microglial elimination. C4 overexpressing mice exhibit decreased spine density in the brain, increased microglial engulfment of synapses (Yilmaz et al., 2021), and altered mouse behavior. An increase in C4 expression in mice induces excessive synaptic pruning, leading to hyperactivity in brain tissue (Sellgren et al., 2019; Yilmaz et al., 2021). In mice microglia exhibit a less complex, less branched morphology, reduced motility, and reduced synaptic adhesion (Kessels et al., 2025; Shiwaku et al., 2022). The behavioral phenotype associated with SCZ can also be induced by altered actin organization, which is directly involved in microglial cytoskeletal dynamics (Kessels et al., 2025). The disrupted in schizophrenia 1 (DISC1) protein regulates microglia cytoskeletal dynamics, and its deficiency increased expression of genes involved in the cascade that enhanced synaptic elimination (Kessels et al., 2025; Yilmaz et al., 2021; Brennand et al., 2015). To study microglia-specific roles for DISC1, the authors used bone marrow transplantation in which WT or *Disc1^LI/LI^* hematopoietic precursors repopulated the brain of WT or *Disc1^LI/LI^* hosts. The results show that DISC1 regulates microglia behavior by altering cytoskeletal organization, motility, and synaptic phagocytosis. DISC1 deficiency in microglia was sufficient to alter key phagocytic (e.g., increased uptake of synaptic material, such as postsynaptic density 95 [PSD95]) and functional properties, independent of neuronal influence. Disc1 WT microglia repaired cognitive and behavioral deficits in *Disc1^LI/LI^* mice (Kessels et al., 2025).

The growing evidence indicates that schizophrenia involves an imbalance between immune dysfunction, synaptic structural alterations, and functional changes in microglia. Genetic factors, such as the *C4* gene and its isoform alterations, can promote excessive synaptic pruning, reduce synaptic density, and contribute to SCZ-related behaviors (Yilmaz et al., 2021; Fournier et al., 2024; Sellgren et al., 2019). An uncommon form of excessive microglial synaptic pruning is considered to be a key mechanism underlying the structural and behavioral phenotype in schizophrenia.

Other factors besides modification in microglia structure contribute to schizophrenia pathology. Experimental data demonstrated synaptic loss as a central pathological hallmark of SCZ, potentially linked to activated microglia and increased inflammatory processes in glial cells (Breitmeyer et al., 2023; Koskuvi et al., 2024). In iPSC-derived microglial cells from SCZ patients, RNA sequencing identified microglial activation, upregulation of several inflammatory genes, including *NNAT, GSTM, and NLRP2*, increased TNFα secretion, and elevated NFκB signaling compared with healthy microglia. This was associated with upregulation of the inflammasome genes *NLRP2* and *NLRP3,* which are targets of NFκB signaling and are involved in caspase-1-mediated inflammasome activation (Breitmeyer et al., 2023). Kosruvi et al. also reported increased expression of genes involved in inflammatory processes (*HLA-DRA* and *IL1β*) in iPSC cells from individuals with SCZ (Koskuvi et al., 2024). Analysis of the microglial activation marker TSPO (a translocator protein 18 kDa) in patients with schizophrenia symptoms persisting longer than 10 years and healthy controls showed a positive correlation of TSPO with behavioral symptoms such as apathy and isolation. The patient’s age was an important factor, with greater TSPO expression in older individuals than in younger individuals, regardless of diagnosis (Conen et al., 2021). These results suggest that TSPO levels, such as microglial hyperactivation, may be a marker for pathogenic mechanisms in schizophrenia. Sex differences can impact SCZ. Rim et al. reported that male mice are more susceptible to the development of SCZ-relevant behaviors than females after prenatal dexamethasone treatment, and that microglia from males exhibit greater synaptic elimination, lower microglial density, and a more branched morphology (Rim et al., 2022).

**Figure 3:**
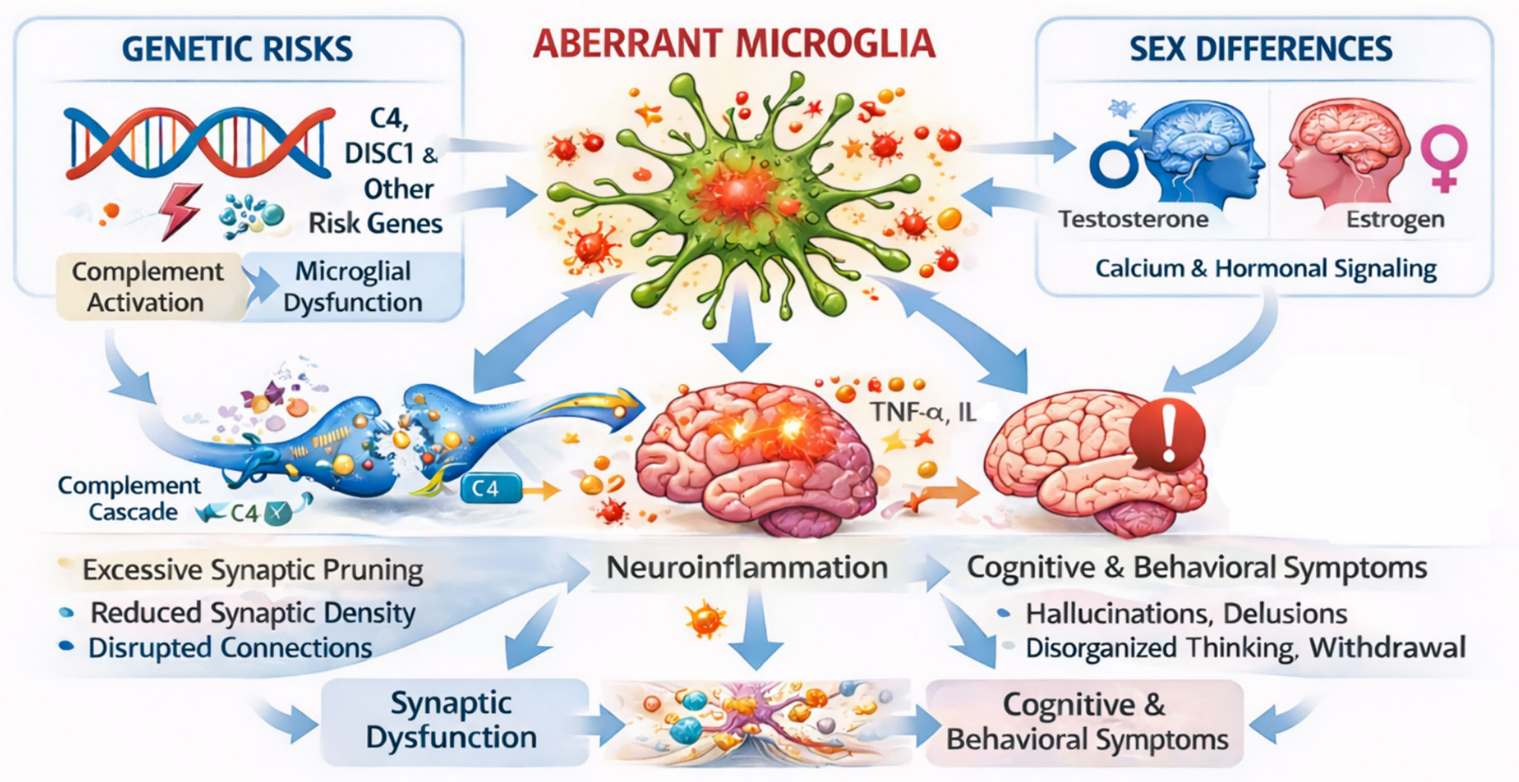
Mechanisms of aberrant microglial function in schizophrenia.

Summarizing, the evidence supports an important role of microglial dysfunction/aberrant activation in the pathology of schizophrenia. This includes genetic factors, immune dysregulation, and synaptic abnormalities. Some alterations, such as the *C4* and *DISC1* genetic variants, lead to excessive synaptic pruning and disrupted neuronal connectivity. These effects are associated with a pro-inflammatory state of microglia, which, through released cytokines, affects neuronal metabolism, mitochondrial function, and the redox balance between microglia and other CNS cells (Figure 3).

Genetic risk factors, such as *C4* isoforms and *DISC1* deficiency, promote abnormal microglial activation, excessive synaptic pruning, and reduced synaptic density. These molecular alterations are related to actin organization, microglial motility, changes in synaptic adhesion, and inflammation. In parallel, sex-dependent factors can affect microglial behavior through hormonal regulation and calcium channel signaling. Aberrant microglial activity also induces neuronal metabolic dysfunction, which includes mitochondrial impairment and oxidative stress. All these alterations result in cognitive and behavioral symptoms characteristic of schizophrenia.

### 2.5 Sex-dependent differences in microglial action in psychiatric disorders

Sex differences are recognized as one of the fundamental variabilities in neuroimmune regulation and could be critical determinants of microglial function in both physiological and pathological contexts. Microglia exhibit sexually dimorphic developmental trajectories, transcriptional profiles, and responsiveness to hormonal and environmental cues, which together shape brain circuit maturation and vulnerability to disease. These differences are particularly relevant in psychiatric disorders, in which prevalence, symptoms, and treatment responses often differ significantly between males and females, suggesting that sex-specific microglial mechanisms may contribute to disorder-specific pathophysiology.

Microglia exhibit profound sex-dependent responses to stress and genetic manipulation, which correspond to differences in susceptibility to affective disorders (Wohleb et al., 2018; Wei et al., 2014; Garrett and Wellman, 2009; Mahmoud et al., 2016; Bollinger et al., 2016). Female RKO mice show a 10-fold increase in pro-inflammatory cytokine expression compared to a 2-fold increase in males (Chen et al., 2023). Microglia depletion with PLX5622 almost completely abolished neuroinflammation in RKO males, whereas the effect was much weaker in females (Chen et al., 2023). In the CUS (chronic unpredictable stress) model, male mice show higher microglial CSF1R expression and a significant reduction in dendritic spine density compared with females, who are often more resilient to stress-induced neuronal impairments (Wohleb et al., 2018). Prenatal exposure to dexamethasone (DEX) also triggered gender-specific remodeling of microglial processes in the PFC (Caetano et al., 2017). This sexual dimorphism is also noticeable in resistance to therapeutic intervention in females, as a selective adenosine A2A receptor (A2AR) antagonist normalized behavior and microglia only in DEX-treated males (Caetano et al., 2017). ScRNA-seq revealed differential expression in the microglia of depressed females who committed suicide, but not in males (Li et al., 2021; Wu et al., 2023). Altered microglial transcription was found in MDD females versus controls, with pro-inflammatory (interferon and NF-KB signaling) and anti-inflammatory (IL4, IL13, and IL10 signaling) pathways downregulated. Genes related to “neuronal” pathways (such as neurotransmitter signaling and ion channels) were upregulated in female MDD microglia (Maitra et al., 2023). WGCNA (Weighted Gene Co-expression Network Analysis) results pointed to downregulation of communication of Parvalbumin immunoreactive interneurons to microglia via GAS6-MERTK signaling and upregulation of SPP1 (secreted phosphoprotein 1) to integrin signaling in the opposite direction in females with MDD (Maitra et al., 2023).

The genetic setting of MDD reveals a higher burden of genetic risk in females. This is characterized by increased polygenicity and the presence of female-biased risk variants that impact microglial function (Thomas et al., 2025). Female-specific susceptibility to MDD is potentially linked to causal risk variants mapping to microglial immune signaling genes coding for TLR4, CYSTM1, PFDN1, SLC4A9, and RAB27B, as well as specific female-biased loci such as GPC6 and SHISA9 (Fitzgerald et al., 2024; Thomas et al., 2025). Furthermore, a higher fetal microglial PGS (polygenic scores) in females moderates the impact of prenatal maternal depressive symptoms on adult outcomes, emphasizing that microglial pathways are central to the sex-dependent translation of early environmental exposures into psychiatric risk (Fitzgerald et al., 2024). Sex-specific microglial adaptations to chronic unpredictable stress (CUS) reveal a distinct molecular dichotomy within the medial prefrontal cortex (mPFC). In males, CUS promotes a transient shift toward a reactive microglial phenotype characterized by the upregulation of CSF1R and apolipoprotein E (APOE), alongside increased expression of the phagocytic marker CD68 (Woodburn et al., 2021). This male-specific microglial engagement is driven by the induction of neuroimmune signals such as CSF1 and IL-34 (Woodburn et al., 2021). Female microglia remain relatively unresponsive during early stress stages, maintaining a higher expression of homeostatic markers and showing the reduced ratio of broadened-to-surveillant morphology (Woodburn et al., 2021; Bollinger et al., 2016). Females also show increased levels of prefrontal *Cx3cl1* and *Cx3cr1* transcript alongside greater *Csf1r, Cd11b,* and *Tgfbr1* expression in frontal cortex microglia (Bollinger et al., 2016; Woodburn et al., 2021). These gene pathways are particularly relevant to microglia–neuron interactions and are important regulators of microglial survival, migration, and synaptic pruning (Schafer et al., 2012; Elmore et al., 2014; Paolicelli et al., 2011). Moreover, female-specific regulators like the long non-coding RNA *FEDORA* are uniquely overexpressed in the prefrontal cortex of women with depression, while prolonged stress in females leads to a specific increase in microglial *TNFα* levels, potentially serving as a compensatory mechanism to maintain neuroimmune stability (Woodburn et al., 2021; Hodes et al., 2024).

Regional microglial heterogeneity and transcriptomic signatures of senescence display profound sexual dimorphism under homeostatic conditions, predisposing individuals to divergent psychiatric outcomes. Female microglia across the prefrontal cortex, striatum, and midbrain exhibit consistently higher expression of selenium-related transcripts, including *Selenow, Selenom, Selenoh,* and *Gpx1,* which play crucial roles in microglial antioxidant protection and metabolic regulation (Barko et al., 2022). In the aging hippocampus, female microglia more robustly adopt senescent and disease-associated signatures compared to males, characterized by the upregulation of *Cd74, Gpr34,* and genes coding for major histocompatibility complex II (MHCII) components such as RT1-Ba, RT1-Bb, and RT1-Db1 (Ince et al., 2023; Ocañas et al., 2023). In contrast, male microglia maintain a more homeostatic transcriptome during brain aging but exhibit distinct inflammatory profiles in response to specific insults, such as the preferential upregulation of the *Irf7* and *Ifit1* pathways (Ince et al., 2023; Barko et al., 2022). These findings indicate that while male microglia are more sensitive to acute inflammatory priming, female microglia are more prone to chronic, age-associated functional decline (Ince et al., 2023; Ocañas et al., 2023).

Also in ASD there is evidence of sex-specific differences in microglia action during the disease development and progression. ASD is diagnosed four times more often in males than females, though this varies by age and IQ (Napolitano et al., 2022). Mutations in genes coding for the Neuroligin (NLGN)-3 and (NLGN)-4 have been associated with intellectual disability and ASD. Guneykaya et al. showed that male NLGN4^−^/^−^ microglia in the hippocampal CA3 region display reduced density, a less ramified morphology compared to male wild-type controls (Guneykaya et al., 2023). In contrast, microglia in female NLGN4^−^/^−^ mice were much less affected in these structural parameters. Transcriptomic and proteomic studies showed higher expression of MHCI and MHCII in male cortical and hippocampal microglia, and higher baseline outward and inward currents, as well as a higher response to ATP in a P2X-dependent manner, measured in cortical slices. Expression of P2X4, P2X7, and P2Y12 receptors in whole-brain microglia was higher in males (Guneykaya et al., 2018; Guneykaya et al., 2023). The observed sex-related differences in key cellular functions and parameters in microglia suggest that hormonal modulation can alter the microglial phenotype in ASD.

Sex-associated dimorphism of microglia function also impacts SCZ. Rim et al. reported that male mice are more susceptible to the development of SCZ-relevant behaviors than females after prenatal dexamethasone treatment. That microglia from males exhibit greater synaptic elimination, lower microglial density, and a more branched morphology (Rim et al., 2022). The hyper-branched phenotype of male microglia in SCZ facilitates excessive synaptic pruning of proteins such as PSD95 in parvalbumin interneurons (PV), which is directly related to the loss of synaptic density (Fournier et al., 2024). Overexpression of the C4 gene has shown that male and female brains respond in divergent ways. While males exhibited hypoexcitability of PV cells and pyramidal neurons, females exhibited hyperexcitability of PV cells. Fournier et al. also demonstrated that synaptic dysfunction in male has alterations in synaptic connections in the medial prefrontal cortex, characterized by a reduction in excitatory stimuli and an increase in inhibitory inputs.

Female hormones can also positively modulate genes involved in peripheral calcium signaling, such as *ATP2B2*, indicating distinct mechanisms between males and females in disease progression (Taheri et al., 2025). Microglia from females showed an inflammatory phenotype with increase in the IL-1β, IL-6, and TNF-α levels in the hippocampus and activation of the hypothalamic–pituitary–adrenal axis (Rim et al., 2022). Moreover, overexpression of the *CACNB4* gene reduced the density of small dendritic spines in females through variation in the interactome of the β4 subunit of the calcium channel. On the other hand, males showed an enrichment of the β1b subunit, which could be a protective factor against synaptic loss (Paolicelli et al., 2022).

Provided evidence indicates that microglial sex-dependent heterogeneity is a key modulator of neuroimmune signaling, synaptic remodeling, and circuit-level dysfunction across psychiatric disorders. These differences are reflected at multiple levels, including transcriptional regulation, receptor expression, cellular morphology, and functional interactions with neurons and other glial populations. The directionality of these effects is specific for different disorders, with males often exhibiting enhanced susceptibility in ASD and SCZ models, while females show distinct inflammatory and transcriptional signatures in MDD. This highlights the necessity of incorporating sex as a biological variable in both experimental design and therapeutic development, as targeting microglial pathways may require sex-tailored strategies to achieve optimal efficacy.

## 3 Conclusion

Advances in single-cell studies, fate lineage tracing and genetic manipulations demonstrated that microglia are long-lived, highly plastic myeloid cells of the CNS that integrate genetic, environmental, and metabolic signals to maintain brain homeostasis. Their dysregulation critically contributes to development and progression of neuropsychiatric disease. Microglia exhibit diverse transcriptional and functional phenotypes shaped by developmental stage, sex, circadian rhythms, stress, immune activation, and local neuronal activity, challenging the notion of a uniform “activated” phenotype. Evidence derived from affective disorders, autism spectrum disorder, and schizophrenia indicates that aberrant microglial signaling drives maladaptive synaptic pruning, altered neurotrophic support, inflammatory and metabolic stress, and circuit-level dysfunction in brain regions affected by the disease. In depression and anxiety, chronic stress, glucocorticoid dysregulation, and peripheral inflammation bias microglia toward pro-inflammatory and phagocytic states that disrupt glutamatergic signaling and plasticity, with strong modulation by the sex and circadian rhythm. In ASD, microglia adopt distinct activation programs characterized by dysregulated immune signaling, epigenetic remodeling, and excessive or mistimed synaptic elimination, contributing to impaired network connectivity and changes in social behavior. In schizophrenia, genetic risk factors link C4 and DISC1 to aberrant microglial cytoskeletal dynamics, synapse engulfment, and inflammatory pathways, linking immune dysfunction to synaptic loss and cognitive deficits. Together, these findings position microglia as central effectors that integrate immune, genetic, and environmental risk factors to synaptic and behavioral pathology, and highlight the presence of both shared and disorder-specific microglial pathways. Some of those targets may prove to be promising candidates for therapeutic intervention across the neuropsychiatric disease spectrum in the future.
